# Efficacy of Botulinum Toxin A for the Management of Melasma: A Split‐Face, Randomized Control Study

**DOI:** 10.1111/jocd.70376

**Published:** 2025-08-01

**Authors:** Sasipim Chaijaras, Suphagan Boonpethkaew, Sonphet Chirasuthat, Nawara Sakpuwadol, Tanat Yongpisarn, Pimsiri Anansiripun, Vasanop Vachiramon

**Affiliations:** ^1^ Division of Dermatology Faculty of Medicine Ramathibodi Hospital, Mahidol University Bangkok Thailand; ^2^ Division of Dermatology School of Medicine, Walailak University Nakhon Si Thammarat Thailand

**Keywords:** chloasma, hyperpigmented disorder, injectable, neurotoxin, skin quality

## Abstract

**Background:**

Melasma management remains challenging due to its multifactorial nature pathogenesis and recurrent nature. Previous studies showed positive effects of botulinum toxin A (BoNT‐A) for treating and preventing ultraviolet‐induced hyperpigmentation.

**Objective:**

To evaluate the effectiveness of adjunctive incoBoNT‐A injection combined with triple combination cream (TCC, 4% hydroquinone, 0.05% tretinoin, and 0.01% fluocinolone acetonide) for treating and preventing melasma recurrence compared to topical therapy alone.

**Methods:**

A split‐face study was conducted in 30 female patients with melasma. One side of the face was randomly applied TCC to the melasma‐affected areas for 12 weeks (monotherapy), while the contralateral side received TCC and intradermal incoBoNT‐A at baseline and week 12 (combination therapy side). Evaluations were performed at baseline and 2, 4, 8, 12, 16, 20, and 24 weeks. Clinical improvement and melanin index were assessed using the MASI score on the malar area (MASI_m_), and Colorimeter respectively. Patient satisfaction was also evaluated.

**Results:**

Twenty‐eight subjects completed the study. The combination therapy side showed significant MASI_m_ decrease at week 2 (*p* = 0.0032), while the monotherapy side showed no significant change. At 4 weeks, a greater reduction of MASI_m_ was observed in the combination therapy side (MASI_m_ 14.5 and 11.54, 20.41% reduction) when compared to the monotherapy side (MASI_m_ 11.68 and 11.79, 0.93% worsening). At week 12, worsening of melasma was observed on both sides during the summer period. At week 24 (3 months after discontinuing TCC), MASI_m_ was 14.79 on the monotherapy side (worsen 21.03% from baseline) and 9.14 on the combined technique (36.97% improvement, *p* = 0.0003). Patients' satisfaction was higher for the combination therapy when compared to the monotherapy at the end of the study (8.92 vs. 7.04, *p* < 0.0001). No serious adverse events occurred.

**Conclusion:**

Intradermal incoBoNT‐A injection combined with TCC demonstrated superior efficacy in melasma treatment and recurrence prevention compared to TCC monotherapy.

## 1 Introduction

Melasma is a common acquired hyperpigmentary disorder characterized by symmetrical brown or grayish‐brown patches on sun‐exposed areas, especially the face. It most frequently affects individuals with darker skin types (Fitzpatrick skin type III‐V), particularly those of Asian, Middle Eastern, Mediterranean‐African, and Hispanic African descent [1, 2, 3]. The exact pathogenesis of melasma involves an interplay of various factors, including genetic predisposition, ultraviolet (UV) radiation, heat, hormonal influences, and inflammation [1, 4]. Histologically, melasma is characterized by increased melanin deposition in the epidermis and/or dermis along with changes in the dermis (i.e., increase mast cell, solar elastosis, hypervascularization, etc.) [5, 6]. These changes result in the increased melanogenic activity of melanocytes in melasma.

Current treatment options for melasma include topical medications that inhibit melanin synthesis, melanin transfer, or increase epidermal turnover (e.g., hydroquinone, tretinoin, corticosteroids, azelaic acid, arbutin, niacinamide, glycolic acid, etc.) as well as procedural therapies (i.e., chemical peels and various lasers) [7]. However, melasma remains challenging to treat, with high recurrence rates [8].

Botulinum toxin A (BoNT‐A) is a potent neurotoxin derived from *Clostridium botulinum* that inhibits acetylcholine release at the neuromuscular junction. It is widely used for both therapeutic and cosmetic indications [9]. Recent studies suggest that BoNT‐A may have additional benefits beyond its effects on muscle activity. According to Jung et al., BoNT‐A exhibited anti‐melanogenic properties and prevented UVB‐induced hyperpigmentation in animal models, partly by inhibiting tyrosinase activity [10]. A clinical study also demonstrated decreased skin pigmentation at 15 days after injection of onaBoNT‐A in the face for wrinkles [11].

Most recently, two randomized controlled trials by Vachiramon et al. provided strong evidence for the efficacy and safety of incoBoNT‐A in treating UVB‐induced hyperpigmentation. Intradermal injection of incoBoNT‐A resulted in significantly reduced pigmentation compared to normal saline and no treatment, based on both clinical and histopathological assessments [12, 13].

Given the promising findings on incoBoNT‐A for treatment of UVB‐induced hyperpigmentation and the need for more effective therapies for melasma, the aim of this study was to evaluate the efficacy and safety of incoBoNT‐A injections as an adjunctive treatment for melasma. We hypothesized that combining incoBoNT‐A with triple combination cream (TCC) would lead to greater improvements in melasma severity and lower recurrence rates compared to TCC alone.

## 2 Materials and Methods

### 2.1 Type of Study

This study was a prospective, split‐face, randomized controlled trial aimed to evaluate the efficacy of incoBoNT‐A as an adjunctive treatment for melasma compared with standard therapy alone. The study protocol was approved by the Institutional Review Board of the Faculty of Medicine Ramathibodi Hospital (Protocol number MURA 2023/480). All participants provided written informed consent before enrollment. The study was registered at www.thaiclinicaltrials.org as TCTR 20240721002.

### 2.2 Patients

Thirty participants were recruited from the university‐based dermatology outpatient clinic (Ramathibodi Hospital, Mahidol University, Bangkok, Thailand). The inclusion criteria were age ≥ 18 years; no serious medical conditions; clinical diagnosis of mixed type melasma with symmetrical involvement of both malar areas; and willingness to comply with the study protocol and follow‐up visits. Exclusion criteria included active skin infection or inflammation at the treatment sites; open wounds at the treatment sites; known allergy to study medications; neuromuscular disorders; uncontrolled acne vulgaris; history of recurrent herpes infection; use of topical melasma treatments within 1 month or systemic/injectable treatments within 3 months; facial procedures (laser, dermabrasion, peeling, radiofrequency, microfocused ultrasound) within 6 months; facial botulinum toxin injections within 6 months; pregnancy or lactation; and refusal or request for withdrawal from the study.

### 2.3 Calculation of the Sample Size and Randomization

The sample size population was not calculated. Patients were included consecutively during the study period. Each participant's face was randomly assigned to two treatment sides using a computer‐generated block randomization list.

### 2.4 Interventions

One malar area was randomly received a TCC (4% hydroquinone, 0.05% tretinoin, and 0.01% fluocinolone acetonide), which was applied nightly for 12 weeks (monotherapy side). The contralateral side received a similar TCC application with additional intradermal injections of incoBoNT‐A (Xeomin; Merz Pharmaceuticals GmbH, Raleigh, NC) at baseline and week 12. IncoBoNT‐A 100 units was reconstituted with preservative‐free normal saline 2.5 mL (concentration of 1:2.5) and injected intradermally using a 30‐gauge needle (4 mm depth). The approximate dosage is 0.1 mL (4 units) for 4 injection points (i.e., 1 unit/injection point), 1 cm apart, with a total dose of 20–40 units covering the entire melasma patch on the cheek. The injection was administered by board‐certified dermatologists. Furthermore, participants were instructed to avoid sun exposure, apply broad‐spectrum sunscreen with SPF ≥ 50 daily, and not to use any other melasma treatments during the study.

### 2.5 Outcome Measurement

Efficacy assessments were performed at baseline and 2, 4, 8, 12, 16, 20, and 24 weeks. The primary efficacy outcome was the percentage change in the modified Melasma Area and Severity Index (MASI) score for the malar region (MASI_m_). The MASI_m_ was calculated as the area of involvement (A; 0–6 scale) and the sum of pigmentation darkness (D; 0–4 scale) and homogeneity (H; 0–4 scale): MASI_m_ = A × (D + H). A blind dermatologist evaluated standardized photographs using the Visia complexion analysis system (Visia, Canfield Scientific Inc., NJ). Melasma relapse was defined as a ≥ 50% increase in MASI_m_ from week 4.

**FIGURE 1:**
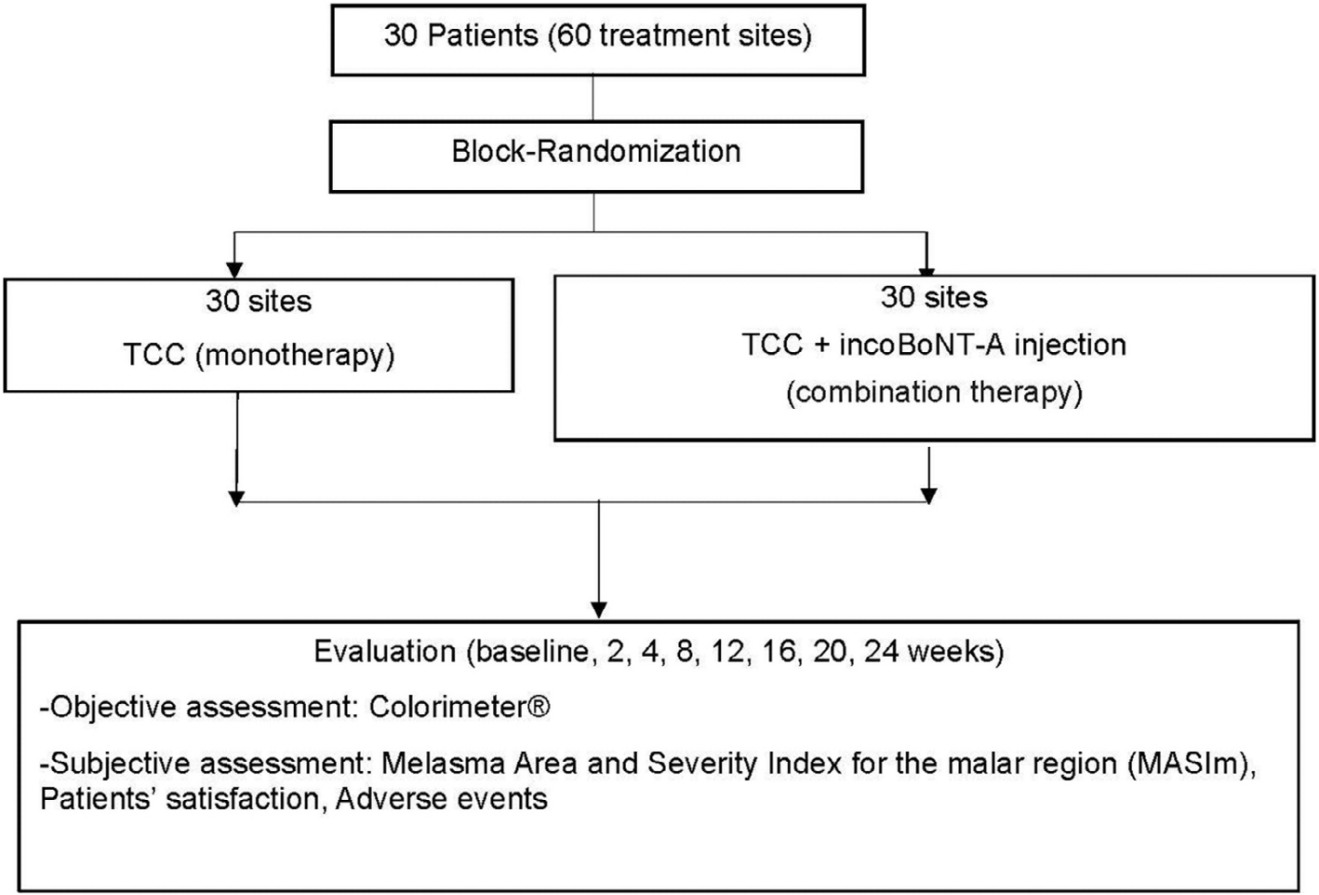
Protocol flow chart.

Other efficacy measures included CIE L* value on Colorimeter (DSM II Colormeter, Cortex Technology, Denmark). Participants rated their satisfaction at the end of the study on a 10‐cm visual analog scale (VAS; 0 = very dissatisfied, 10 = very satisfied). Safety was assessed by monitoring adverse events. The protocol flow chart is demonstrated in Figure 1.

### 2.6 Statistical Analysis

Descriptive data was presented as number (percentage) and mean (standard deviation (SD)) as appropriate. The repeated‐measures ANOVA was used to compare the statistical differences in the means of multiple comparisons (MASI score, CIE L*, and VAS). GraphPad Prism version 10.2.2 was utilized for all statistical analyses and graph presentations. Statistical significance was set at *p* < 0.05.

## 3 Results

Twenty‐eight subjects completed the 24‐week study. Two participants were excluded from the study due to an inconvenient follow‐up schedule. The participants were all female, with a mean age of 43.82 (±8.51). Fitzpatrick skin type III (39.3%), and the other had skin type IV (60.7%). The duration of melasma was 4.93 (±2.90) years, and the average duration of sun exposure was 2.50 (±1.96) hours per day (Table 1).

**TABLE 1:** Demographic data.

Data	Value
Mean age, year (SD)	43.82 (±8.51)
Sex, Female, *n* (%)	28 (100%)
Fitzpatrick skin type, *n* (%)	
III	11 (39.3%)
IV	17 (60.7%)
Duration of melasma, year (SD)	4.93 (±2.90)
Duration of sun exposure, hours/day (SD)	2.50 (±1.96)

**FIGURE 2:**
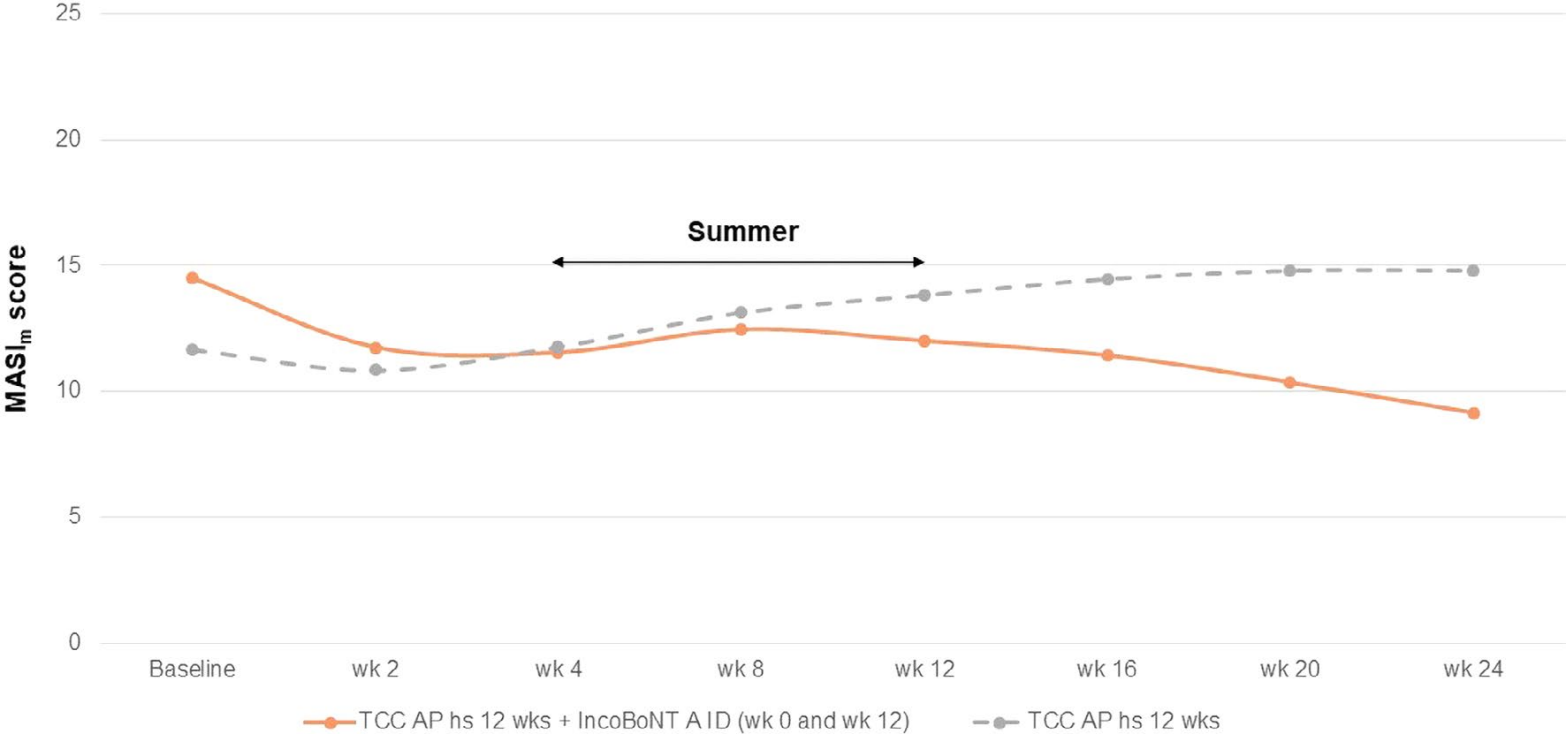
Melasma Area and Severity Index score for the malar region (MASI_m_) at baseline and each follow‐up visit.

In terms of clinical improvement, the mean MASI_m_ at baseline, week 2, week 4, week 12, are 14.50, 11.75, 11.54, 12.00 and 11.68, 10.86, 11.79, 13.82 for combined therapy and monotherapy, respectively. Compared to baseline, a significant improvement was observed in the combination side since week 2 (*p* = 0.0032), while the monotherapy side showed some improvement at week 2 but no statistical significance when compared to baseline. A greater reduction in MASI_m_ was observed in the combination therapy compared to the monotherapy at 4 weeks (20.41% improvement vs. 0.93% worsening) (Figure 2). At week 12, a worsening of melasma was observed on both sides during the summer period.

Patients were instructed to apply TCC for 12 weeks and stop. In the combination group, the patient was treated with incoBoNT‐A at week 12. At the 24‐week follow‐up, the mean MASI_m_ in the combination side and monotherapy side was 9.14 and 14.79, respectively (Figure 2).

Regarding the melanin index, the combination therapy side demonstrated a significant decrease of 3.45% at week 4 compared to baseline (*p* = 0.0068). A reduction of 2.81%, 3.90%, and 5.03% was noted at the 16, 20, and 24 weeks follow‐up sessions after discontinuing TCC (*p* = 0.0105, *p* = 0.0007, *p* < 0.0001, respectively). In contrast, the melanin index persistently increased after discontinuing TCC in the monotherapy side. The combination therapy side demonstrated a higher percentage change in skin lightness (CIE L* value) compared to the monotherapy side throughout the study period.

**FIGURE 3:**
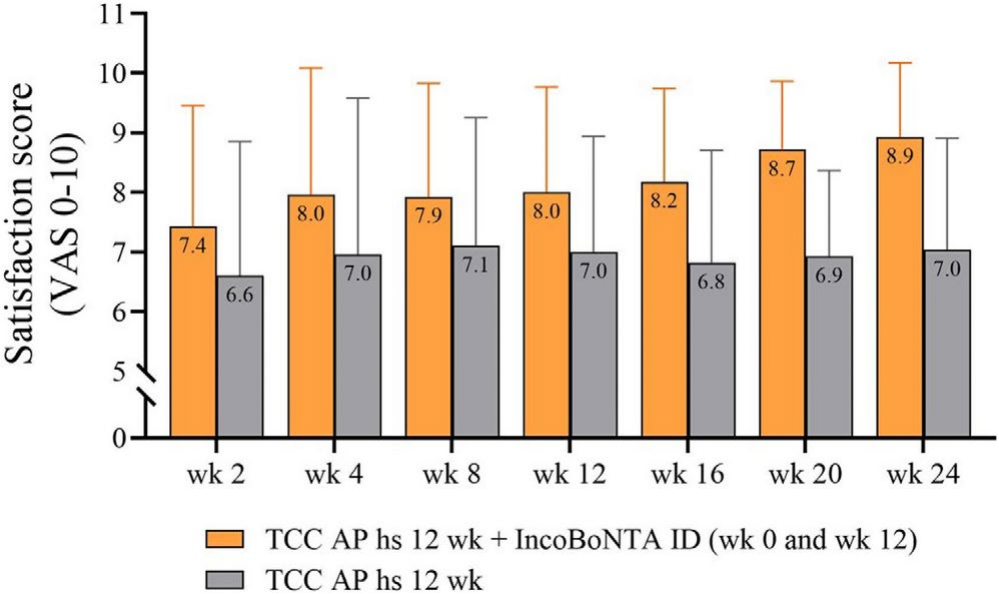
Patient satisfaction score.

**FIGURE 4:**
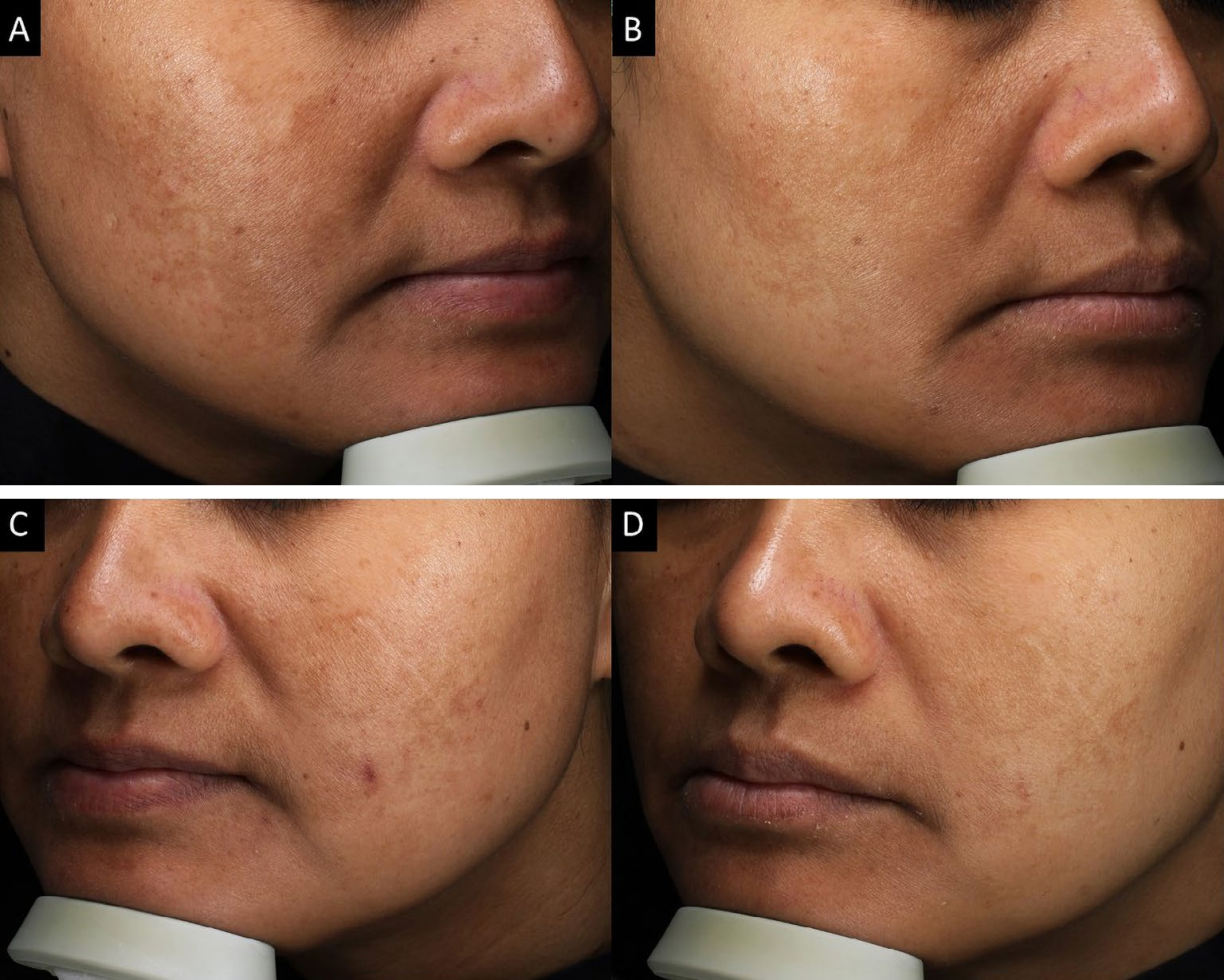
The images show the split‐face treatment sides of the same patient in this study. Baseline and week 24 on combination (A, B) therapy side and monotherapy side (C, D).

In terms of patient satisfaction, a higher satisfaction score in the combined group was observed in all follow‐up visits. At the end of the study (week 24), a significantly higher satisfaction score was observed in the combination therapy compared to the monotherapy (8.92 vs. 7.04, *p* < 0.0001) (Figure 3). The clinical photographs are demonstrated in Figure 4A–D.

No serious adverse events were reported in this study. Mild transient adverse effects, such as erythema and edema at the injection sites, were observed in the combination therapy side and resolved within a few days without additional treatment. No muscle paralysis or asymmetrical smile was observed.

## 4 Discussion

This prospective, randomized, split‐face study demonstrated that the combination of incoBoNT‐A injection and TCC application was more efficacious than TCC alone for the treatment of melasma. The combination therapy resulted in a greater reduction in MASI_m_, higher patient satisfaction, and continuous improvement after discontinuation of TCC. Melasma recurrence was not observed after discontinuation of TCC when compared to TCC monotherapy.

The anti‐melanogenic effects of BoNT‐A have been observed in in vitro studies. Treatment with botulinum toxin A significantly reduced melanin synthesis and tyrosinase activity in cultured human melanocytes [10, 14, 15, 16]. The hypothesis of the anti‐melanogenic effects of BoNT‐A has also been attributed to its ability to inhibit the release of acetylcholine from melanocytes. Acetylcholine has been shown to stimulate melanin synthesis and dendrite formation in melanocytes through the activation of muscarinic receptors. By blocking the release of acetylcholine, BoNT‐A may reduce melanocyte activity and melanin production [10, 17, 18]. Furthermore, BoNT‐A has been found to modulate the expression of various cytokines and growth factors involved in melanogenesis and inflammation (e.g., basic fibroblast growth factor, interleukin‐1α, prostaglandin E2). These cytokines have been implicated in the pathogenesis of pigmentary disorders, including ultraviolet (UV)‐induced hyperpigmentation and melasma, by stimulating melanocyte activity and melanin synthesis [10, 19].

In addition to its direct effects on melanocytes, BoNT‐A may also indirectly influence melanogenesis through its actions on the cutaneous vasculature. BoNT‐A has been shown to inhibit the release of vasoactive substances, such as substance P and calcitonin gene‐related peptide (CGRP), from sensory nerves [20]. These neuropeptides are known to induce vasodilation [21]. By reducing the release of these neuropeptides, BoNT‐A may help to alleviate the vascular component of melasma.

Our findings are consistent with previous clinical studies demonstrating the efficacy of incoBoNT‐A for the treatment of UVB‐induced hyperpigmentation. In a randomized controlled trial, intradermal injection of incoBoNT‐A significantly reduced UV‐induced hyperpigmentation compared to sodium chloride and no treatment [13]. Another study demonstrated that pretreatment with incoBoNT‐A before UVB exposure could prevent the development of hyperpigmentation [12].

According to a recent split‐face study by Thanasarnaksorn et al., intradermal abobotulinum toxin A (aboBoNT‐A) demonstrated a beneficial effect on melasma in 12 patients. The data from the in vitro study also show an inhibitory effect of UVA‐induced melanin content in B16F10 cells [14]. To our knowledge, the present study is the first to investigate the use of incoBoNT‐A for the treatment and prevention of melasma recurrence.

The combination side in our study also exhibited better maintenance of improvement after TCC discontinuation at week 12, suggesting a potential role for incoBoNT‐A in preventing melasma recurrence. This is particularly important given the chronic and relapsing nature of melasma, which often requires long‐term treatment [22]. By targeting the underlying pathogenic mechanisms, such as UV‐induced melanogenesis and inflammation, BoNT‐A may be a potential tool to break the cycle of melasma recurrence and reduce the need for continuous topical therapy.

The safety profile of incoBoNT‐A in this study was favorable, with only mild and transient injection site reactions reported. This is consistent with the well‐established safety record of BoNT‐A in various dermatological indications [23]. However, BoNT‐A neutralizing antibody may occur with the intradermal technique, similar to intradermal vaccination [24, 25]. Therefore, the use of incoBoNT‐A, a highly purified neurotoxin free of complex proteins, may further minimize the risk of immunogenicity and treatment resistance [26].

The use of BoNT‐A for the management of melasma represents a novel approach that may emerge as a promising option in the future. As melasma remains a challenging condition to treat, with high rates of recurrence and limited long‐term efficacy of current therapies, there is a clear need for innovative strategies. Furthermore, the potential synergistic effects of BoNT‐A with other established treatments, such as topical agents and laser therapy, may open up new avenues for combination therapy and personalized treatment approaches.

Limitations of this study include the small sample size, short follow‐up duration, and lack of cellular and molecular data to explain the beneficial effects of this modality. Larger, long‐term studies are needed to confirm the efficacy and safety of incoBoNT‐A for melasma and to determine the optimal treatment regimen. Additionally, future studies should investigate the potential synergistic effects of BONT‐A with other melasma treatments, such as oral tranexamic acid and laser therapy.

In conclusion, the combination of incoBoNT‐A injections and TCC demonstrated superior efficacy compared to TCC monotherapy for the treatment of melasma. In addition, the preventive effect of BoNT‐A for the melasma recurrence was demonstrated. This novel approach was well‐tolerated and resulted in high patient satisfaction. IncoBoNT‐A may represent a promising adjunctive treatment for melasma, particularly for patients with recalcitrant disease or frequent relapses. As we continue to explore the multiple biological effects of BoNT‐A, its use in the management of melasma and other pigmentary disorders may become more widespread in the future. Further studies are warranted to validate these findings and explore the long‐term benefits of BoNT‐A in the management of melasma.

## 5 Conclusion

The combination of incoBoNT‐A injections and TCC was more effective than TCC monotherapy for the treatment of melasma. In addition, there is a potential role of BoNT‐A for the prevention of melasma recurrence. This combination therapy side approach resulted in greater reduction of MASI_m_, better maintenance of improvement after discontinuation of topical therapy, and higher patient satisfaction. Larger, long‐term studies are needed to confirm these results and optimize treatment protocols.

## Author Contributions

Sasipim Chaijaras: Conducting patient evaluation, collecting data, writing, and revising the manuscript. Supahagan Boopethkaew: Conducting patient evaluation and collecting data. Sonphet Chirasuthat: Conducting patient evaluation and collecting data. Nawara Sakpuwadol: Conducting patient evaluation and collecting data. Tanat Yongpisarn: Conducting patient evaluation and collecting data. Pimsiri Anansiripun: Conducting patient evaluation and collecting data. Vasanop Vachiramon: Conceptualization, methodology, supervison, reviewing, and revising the manuscript.

## Ethics Statement

Reviewed and approved by Ramathibodi Hospital Mahidol University clinical research ethic committee and was conducted in compliance with Good Clinical Practice (COA. MURA2023/480). Trial register number TCTR 20240721002.

## Consent

The patients in this manuscript have given written informed consent to the publication of their case details.

## Conflicts of Interest

The authors declare no conflicts of interest.

## Data Availability

The data that support the findings of this study are available from the corresponding author upon reasonable request.
